# Effects of different *Forsythia suspensa* leaf drying methods on volatile profile, taste-active compounds, and functional properties of millet-forsythia biscuits

**DOI:** 10.3389/fnut.2026.1832965

**Published:** 2026-05-20

**Authors:** Junling Zhu, Yong Qin, Mengyu Cui, Yufan Shang, Yakun Li

**Affiliations:** 1 School of Food Science and Engineering, Shanxi Agricultural University, Jinzhong, China; 2 Shanxi Province Key Laboratory Cultivation Base of Minor Crops Nutrition and Healthy Food Development, Taigu, Shanxi, China

**Keywords:** antioxidant activity, drying methods, free amino acids, microstructure, millet–*Forsythia suspensa* leaf biscuits, volatile flavor compounds

## Abstract

**Introduction:**

The drying method of *Forsythia suspensa* leaves critically affects the flavor retention and functional quality of millet-*Forsythia suspensa* leaf biscuits. This study aimed to evaluate the influence of different drying techniques on the flavor profile, taste-active compounds, microstructure, and antioxidant capacity of these biscuits.

**Methods:**

Four drying methods–sun drying (SD), vacuum freeze drying (VFD), vacuum drying (VD), and hot air drying (HAD)—were compared. Volatile compounds were analyzed using dichloromethane extraction coupled with GC-MS and principal component analysis (PCA). Aroma and taste profiles were assessed with an electronic nose (E-nose) and electronic tongue (E-tongue). Free amino acids, texture, microstructure (SEM), and antioxidant activities (DPPH and ABTS assays) were also measured.

**Results:**

A total of 86 volatile compounds were identified, with aldehydes, alcohols, and ketones as the major contributors. VFD samples had a 28.4% higher total volatile peak area than HAD samples, with better preservation of C6–C9 aldehydes and aromatic compounds. PCA of E-nose data explained 86.7% of variance and clearly distinguished among drying treatments. Taste analysis showed HAD samples had higher overall taste intensity, while VFD samples preserved taste-active amino acids, including glutamic acid and aspartic acid (18.6% and 14.2% higher than HAD, respectively). Texture analysis indicated HAD increased hardness by 21%, whereas VFD improved crispness and cohesiveness. SEM images revealed that VFD maintained a uniform porous structure, whereas HAD caused structural collapse. VFD samples exhibited the highest antioxidant activity (DPPH 67.29%).

**Discussion/conclusion:**

Differences in quality were attributed to the drying mechanisms. VFD minimized thermal degradation and volatile loss, preserving native compounds and bioactive substances. VD provided intermediate preservation, limiting oxidation but causing some compositional changes. HAD promoted lipid oxidation and Maillard reactions, while SD led to gradual oxidation and volatile loss. Vacuum freeze drying effectively enhanced flavor, umami amino acid content, microstructure, and antioxidant activity, providing valuable guidance for optimizing functional cereal-based product processing.

## 1 Introduction

*Forsythia suspensa* leaves are a traditional medicinal and edible plant resource and have long been used as a folk tea in China (1). Phytochemical and metabolomic studies have shown that the leaves are rich in phenolic acids, flavonoids, lignans, terpenoids, and other bioactive secondary metabolites (2). These constituents are associated with antioxidant and other bioactive functions (1, 3). In addition, recent studies on *Forsythia suspensa* leaf fermented tea have shown that the leaves are rich in volatile aroma components and are being increasingly explored for the development and utilization of functional food products (3, 4). However, the application of *Forsythia suspensa* leaves in baked food systems, such as biscuits, and the effects of processing methods on product quality remain insufficiently explored.

In addition to their individual nutritional and functional properties, the combination of millet and *Forsythia suspensa* leaves in biscuit formulations presents a promising strategy for developing novel functional bakery products. Millet is a nutritious grain characterized by high dietary fiber content, balanced amino acid composition, and low glycemic index, giving it broad potential for the development of health foods, which is widely recognized (5, 6). Meanwhile, *Forsythia suspensa* leaves are rich in polyphenols, flavonoids, and phenylethanoid glycosides, which exhibit strong antioxidant and bioactive properties (2, 7). The incorporation of *Forsythia suspensa* leaves into millet based biscuits not only enhances their nutritional and functional properties, but also imparts unique flavor characteristics that are derived from plant secondary metabolites. Furthermore, interactions between cereal components (such as starch and proteins) and plant polyphenols during processing significantly influence the physicochemical properties, flavor profile, and bioavailability of bioactive compounds (8, 9).

In particular, when incorporated into cereal based baked products, such as millet biscuits, the interactions between Forsythia derived bioactive compounds and the food matrix significantly influence flavor, structure, and functional properties. During drying, plant materials undergo moisture migration, cellular collapse, and various enzymatic and non-enzymatic reactions. These processes lead to systematic alterations in volatile compounds, taste active substances, and functional components. Previous studies have demonstrated that drying methods significantly affect the volatile flavor profile and flavor related characteristics of plant and food materials. For instance, vacuum freeze drying is reported to better preserve floral aroma compounds in osmanthus (10). In faba beans and mulberries, different drying treatments markedly alter volatile composition and antioxidant capacity (11, 12). Similar effects have been observed in aquatic products and edible mushrooms, where drying methods governed the formation and transformation of aldehydes, ketones, and characteristic aroma compounds (13, 14). Furthermore, comparative studies on coffee beans confirm that drying modes play a decisive role in shaping volatile profiles and taste characteristics (15).

In addition to aroma changes, the regulatory effects of drying on microstructure, amino acid composition, and functional activity have received increasing attention. Different drying strategies influence cell wall integrity and pore structure, thereby affecting the release of free amino acids and the retention of antioxidant activity. Such mechanisms have been systematically validated in enoki mushrooms and Boletus species (16, 17). In mulberry leaves, vacuum freeze drying and vacuum microwave drying are shown to better preserve amino acids and bioactive compounds (10). Although these studies have clarified the influence of drying on volatile flavor, microstructure, and functional properties individually, systematic investigations on *Forsythia suspensa* leaves applied in baked food systems remain limited. Moreover, when *Forsythia suspensa* leaves are incorporated into a real baked matrix such as millet biscuits, leaf derived polyphenols are expected to interact with biscuit starch and proteins during dough formation and baking. Previous studies have shown that starch polyphenol and protein polyphenol interactions can occur through covalent or non-covalent associations, and that these interactions may alter polyphenol stability, release behavior, antioxidant expression, and matrix structure. Therefore, the functional and quality outcomes observed in millet biscuits are not expected to be identical to those of isolated leaf materials (8, 9, 18). In particular, the coordinated changes among volatile compounds, taste active substances, and functional quality within a real food matrix are not fully elucidated.

Therefore, in this study, *Forsythia suspensa* leaves subjected to different drying treatments are incorporated into millet biscuits, and all subsequent analyses are performed on the resulting biscuit products. A comprehensive analytical approach is employed, including electronic nose, electronic tongue, GC–MS, texture analysis, and antioxidant activity assessment. The effects of sun drying (SD), vacuum freeze drying (VFD), vacuum drying (VD), and hot air drying (HAD) on volatile composition, taste characteristics, and functional quality are systematically compared. The aim is to elucidate the mechanisms by which drying methods regulate quality formation in *Forsythia suspensa* enriched baked products, thereby providing theoretical support for process optimization and quality improvement of functional bakery foods.

## 2 Materials and methods

### 2.1 Main materials and reagents

Millet, wheat flour, egg, white granulated sugar, baking powder, salt, butter were obtained from a local supermarket in Jinzhong, Shanxi, China. Fresh *Forsythia suspensa* leaves were purchased from the Forsythia Planting Base of Shanxi Agricultural University. Anhydrous ethanol, hydrochloric acid, methanol, petroleum ether, anhydrous sodium sulfate, sodium citrate, sodium hydroxide, potassium hydroxide, anhydrous sodium carbonate, aluminum nitrate non-ahydrate, sodium hydroxide, glacial acetic acid, trichloromethane, potassium iodide, sodium thiosulfate, potassium persulfate, ferrous sulfate, hydrogen peroxide, salicylic acid, 2,2-diphenyl-1-picrylhydrazyl (DPPH), ABTS reagent, rutin standard, amino acid mixture standard mixture were purchased from Beijing Solarbio Science and Technology Co., Ltd. (Beijing, China), unless otherwise specified. All chemicals and reagents used in this study were of analytical grade. Deionized water was used throughout all experiments.

### 2.2 Main instruments and equipment

DHG-9070A Electric Oven (Shanghai Yiheng Scientific Instrument Co., Ltd., Shanghai, China); FW100 Crusher (Tianjin Taist Test Instrument Co., Ltd., Tianjin, China); DNP-9082 Temperature Controlled Incubator (Shanghai Jinghong Experimental Equipment Co., Ltd., Shanghai, China) TA.XTplus Texture Analyzer (Stable Micro Systems, Surrey, UK); C-PEN3 Electronic Nose (Beijing Yingshenghengtai Technology Co., Ltd., Beijing, China); SA402B Electronic Tongue (Insent Inc., Atsugi, Japan); TGL-16M High-Speed Refrigerated Centrifuge (Xiangyi Centrifuge Instrument Co., Ltd., Hunan, China); LA8080 Amino Acid Automatic Analyzer (Hitachi High Tech Corporation, Tokyo, Japan); Regulus 8100 Scanning Electron Microscope (Hitachi High Tech Corporation) and UV-2600 UV-Vis Spectrophotometer (Shimadzu Corporation, Kyoto, Japan) were used in this study.

### 2.3 Test methods

#### 2.3.1 Preparation process of millet–*Forsythia suspensa* leaf biscuits

Wheat flour (40 g) was used as the base material. Millet flour (40%, w/w), *Forsythia suspensa* leaf powder (9%, w/w), granulated sugar (20%, w/w), butter (15%, w/w), and egg (20%, w/w) were added based on the mass of wheat flour. Baking powder (2%, w/w) and salt (1%, w/w) were subsequently incorporated. All dry ingredients were thoroughly blended to ensure uniform distribution. An appropriate amount of water (13 g, corresponding to 32.5% of the wheat flour mass) was gradually added, and the mixture was kneaded until a homogeneous dough with a smooth surface was obtained. The dough was allowed to rest for 5 min at room temperature. It was then repeatedly sheeted and rolled into a uniform thin layer. The sheeted dough was cut into pieces using a mold and arranged evenly on a baking tray. Baking was performed in a preheated oven at 150 °C (top and bottom heat) for 10 min. After baking, the biscuits were cooled to room temperature and sealed in food grade packaging bags. The final product was stored under ambient conditions until further analysis. To serve as a blank control, an additional group of biscuits without *Forsythia suspensa* leaf powder (control biscuits) was prepared by replacing the 9% (w/w) Forsythia leaf powder with an equal amount of millet flour, while keeping all other ingredients and processing steps identical to those described above.

#### 2.3.2 Drying process design

Fresh *Forsythia suspensa* leaves from the same harvest batch were used in this study. Immediately after collection, leaves without mechanical damage or pest infection and with uniform maturity were selected. The samples were washed to remove impurities and surface water was drained. Leaves were spread in a single layer with uniform thickness, and each batch weighed 500 g.

Sun drying (SD): fresh *Forsythia suspensa* leaves were evenly distributed on stainless steel trays and exposed to natural sunlight from 9:00 to 17:00 on consecutive sunny days. During nighttime, samples were transferred to a ventilated indoor environment to prevent moisture absorption. This process was repeated daily until the moisture content decreased to below approximately 8%. Dried samples were then sealed and stored for subsequent analysis.

Hot-air drying (HAD): fresh *Forsythia suspensa* leaves were spread uniformly on trays in a hot air circulation oven. Drying was conducted at 60 °C with an air velocity of approximately 1.5 m/s under continuous operation. Samples were manually turned every hour to ensure uniform heat distribution. Drying continued until the moisture content reached below approximately 8%. After drying, samples were cooled to room temperature and sealed for storage.

Vacuum drying (VD): fresh *Forsythia suspensa* leaves were placed in a vacuum drying oven at 50 °C. The vacuum pressure was maintained at −0.08 MPa throughout the process. No additional operational parameters were adjusted during drying. The treatment was terminated when the moisture content fell below approximately 8%. Dried samples were cooled to room temperature and stored in sealed, light-protected conditions.

Vacuum freeze-drying (VFD): fresh *Forsythia suspensa* leaves were prefrozen at −40 °C for 12 h to ensure complete solidification. The frozen samples were then transferred to a vacuum freeze dryer for sublimation drying. The condenser temperature was set at −65 °C, and the chamber pressure was maintained below 20 Pa. Drying was continued until the moisture content decreased to below approximately 8%. Samples were promptly sealed after removal to prevent moisture uptake.

All four drying treatments were controlled using the same moisture endpoint (< 8%), determined according to GB 5009.3-2016 (National Food Safety Standard: determination of Moisture in Foods, direct drying method). This ensured that the observed differences among treatments were attributable solely to the drying conditions, and provided a reliable basis for the subsequent comparison of volatile compounds, taste active substances, and functional quality attributes.

#### 2.3.3 Volatile component analysis

##### 2.3.3.1 Electronic nose analysis

The odor profiles of the samples were determined using an electronic nose, with each group being repeated three times. A 5 g of the sample was accurately weighed and placed in a 40 mL headspace vial. The sample was heated in a 50 °C water bath for 30 min. The operating parameters were as follows: a sampling time of 120 s, a cleaning time of 60 s, and an injection flow rate of 200 mL/min. The specific standards were presented in Table 1.

**Table 1:** Types of sensitive substances corresponding to sensors.

Serial number	Sensor name	Sensitized substance type
1	W1C	Aromatic hydrocarbons
2	W5S	Nitrogen oxides
3	W3C	Ammonia, aromatic components
4	W6S	Hydride
5	W5C	Alkenes, aromatic compounds
6	W1S	Alkanes
7	W1W	Inorganic sulfides
8	W2S	Alcohols and some aromatic compounds
9	W2W	Aromatic compounds and organic sulfides
10	W3S	Alkanes and aliphatic compounds

##### 2.3.3.2 GC-MS analysis

Sample pre-treatment: the millet *Forsythia suspensa* leaf biscuits prepared using leaves subjected to different drying treatments were crushed and passed through a 60 mesh sieve, and then thoroughly mixed. A 5 g portion of the homogenized sample was accurately weighed and placed into a 50 mL capped centrifuge tube. Subsequently, a 20 mL of chromatographic grade dichloromethane was added for volatile extraction. Dichloromethane was selected as the extraction solvent due to its broad extraction capability for volatile compounds with different polarities. An internal standard (octanol, 100 μg/mL, 20 μL) was added for semiquantitative analysis. The mixture was ultrasonicated at room temperature for 30 min (300 W), followed by centrifugation at 5,000 rpm for 10 min. The supernatant was collected, dehydrated with anhydrous sodium sulfate, filtered through a 0.22 μm organic membrane, and transferred into a sample vial for subsequent GC–MS analysis (19–21).

GC conditions: a DB-5MS quartz capillary column (30 m × 0.25 mm × 0.25 μm) was used for chromatographic separation. High purity helium (99.999%) was employed as the carrier gas at a flow rate of 1 mL/min. The injection volume was 1 μL, and samples were introduced using a split injection mode with a split ratio of 10:1. The injection port temperature was set at 250 °C.

Oven temperature program: the initial temperature was set at 40 °C and held for 3 min, then increased to 150 °C at a rate of 5 °C/min, followed by a further increase to 250 °C at 10 °C/min and held for 5 min.

MS conditions: electron ionization (EI) mode was used with an ionization energy of 70 eV. The ion source temperature was set at 230 °C, the quadrupole temperature at 150 °C, and the interface temperature at 250 °C. The mass scan range was m/z 35 to 500, and data were acquired in full scan mode.

#### 2.3.4 Flavor components and amino acid profile

##### 2.3.4.1 Electronic tongue analysis

Sample pre-treatment: to ensure the consistency and homogeneity of sample analysis, the biscuit samples were mixed with deionized water at a ratio of 1:10 (w/v). The mixture was thoroughly homogenized and extracted at room temperature by shaking. Subsequently, the mixture was centrifuged at 5,000 rpm for 10 min, and the supernatant was collected for further analysis. A 35 mL aliquot of the supernatant was accurately transferred into the electronic tongue measurement cups. Three parallel cups were prepared for each sample for the determination of six taste indicators (22, 23).

Determination conditions: before the measurements, the pH value and conductivity of the sample solution were measured to ensure that they met the requirements of the instrument's detection range. The reference solution was prepared using a KCl tartaric acid solution. The negative electrode cleaning solution consisted of a mixture of water, ethanol, and HCl, while the positive electrode cleaning solution consisted of KCl, water, ethanol, and KOH. Prior to analysis, the taste sensor was immersed in the reference solution for 30 s to allow stabilization and calibration. The taste measurements were then performed, with each measurement lasting 30 s. After each measurement, the sensor was rinsed with the reference solution for 3 s before proceeding to the next measurement.

##### 2.3.4.2 Quantitative analysis of free amino acids

Sample pre-treatment: an appropriate amount of the mixed sample (accurate to 0.0001 g) was weighed and placed into a hydrolysis tube. Subsequently, a 5 mL of 1:1 (v/v) hydrochloric acid solution was added, and the mixture was thoroughly mixed, sealed, and hydrolyzed at 110 ± 1 °C for 22 h. After hydrolysis, the sample was cooled to room temperature and filtered into a 10 mL volumetric flask. The hydrolysis tube was rinsed several times with a small amount of deionized water, and the washings were combined and brought to volume with deionized water, followed by thorough mixing. Then, 0.05 mL of the filtrate was accurately transferred into a 15 mL test tube and evaporated to dryness under a stream of nitrogen gas. The residue was reconstituted to 2 mL with 0.02 mol/L hydrochloric acid solution and mixed thoroughly. The solution was subsequently filtered through a 0.22 μm microporous membrane and used for instrument analysis (24, 25).

Analysis conditions: a sulfonic acid type cation exchange resin column was used for chromatographic separation. The injection volume was 20 μL. The detection wavelengths were set at 570 nm for common amino acids and 440 nm for proline. The reaction temperature was maintained at 135 ± 5 °C. Gradient elution was performed using a sodium citrate buffer system, and the flow rate and gradient program were carried out according to the standard analytical method of the instrument. Quantification was conducted using the external standard method, and the content of each amino acid was calculated based on the corresponding standard calibration curves.

#### 2.3.5 Texture and microstructure

##### 2.3.5.1 Texture determination of millet and forsythia cookies

Texture analysis was performed using a TA.XTplus texture analyzer (Stable Micro Systems, Surrey, UK). Samples with intact shapes and similar sizes were selected for measurement. A cylindrical probe (P/5, 5 mm diameter) was used in Texture Profile Analysis (TPA) compression mode. The initial trigger force was set at 0.5 N, the test speed was 1 mm/s, and the strain displacement was 1 mm. During compression, parameters including hardness, fracturability, deformation at break, springiness, and chewiness were obtained from the force–distance curve. These parameters were selected based on commonly reported texture analysis protocols for biscuit and bakery products (15, 26).

##### 2.3.5.2 Observation of microstructure

A biscuit sample was cut into pieces of approximately 5 × 5 × 2 mm, mounted on the sample stage, and dried at room temperature. Subsequently, a gold layer (approximately 10 nm thick) was sputter coated onto the sample to enhance conductivity. The accelerating voltage of the SEM was set at 5–15 kV, and observations were conducted at different magnifications as required. Representative cross sectional images were obtained to characterize the pore structure, fiber network, and particle distribution.

#### 2.3.6 Polyphenols, flavonoids, and antioxidant evaluation

##### 2.3.6.1 Determination of total phenols and total flavonoids in millet–*Forsythia suspensa* leaf biscuits

Total phenol content determination: using gallic acid as the standard substance, a standard curve Y = 2.5191X + 0.0753 (*R*^2^ = 0.9929) was plotted, where X represented the mass concentration (μg/mL) and Y represented the absorbance. 0.4 g of sample powder was weighed and extracted with 70% ethanol solution. After magnetic stirring, ultrasonic assistance and centrifugation, the supernatant was collected (26). The same procedure was applied to the control biscuit samples (without *Forsythia suspensa* leaf powder) prepared as described in Section 2.3.1. The reaction system included the sample solution, the Folin phenol reagent and sodium carbonate solution. The reaction was carried out at room temperature in the dark for 90 min, and the absorbance was measured at 760 nm. The total phenol content of the sample was calculated according to the following formula:


$$\begin{array}{l} Total\;phenol\;content\;\left(\mu mol/g\right)=\frac{c\times V}{m} \end{array}$$

Among them, c represented the total phenol concentration (in μmol/L), V represented the total volume (in L), and m represented the sample mass (in g).

Total flavonoid content determination: using rutin as the standard substance, a standard curve Y = 0.8707X + 0.0461 (*R*^2^ = 0.9925) was plotted, where X represented the mass concentration (mg/mL) and Y represented the absorbance. We weighed 2.5 g of the sample, ultrasonically extracted it with 60% ethanol and then centrifuged to obtain the supernatant. After sampling, it was reacted with sodium nitrite, aluminum nitrate, and sodium hydroxide successively, and the absorbance was measured at 510 nm. The same procedure was applied to the control biscuit samples (without *Forsythia suspensa* leaf powder) prepared as described in Section 2.3.1. The calculation formula for total flavonoid content was:


$$\begin{array}{l} Total\;flavonoid\;content\;\left(mg/g\right)=\frac{A\times V\times C}{m} \end{array}$$

Among them, A represented the measured absorbance, V represented the total volume (in mL), C represented the dilution factor, and m represents the sample mass (in grams).

##### 2.3.6.2 Determination of the antioxidant capacity of millet–*Forsythia suspensa* leaf biscuits

DPPH radical scavenging capacity determination: a 2.5 g sample was weighed and extracted with 60% ethanol using an ultrasonic method. The mixture was then centrifuged, and the supernatant was collected. The sample solution was mixed with the DPPH solution and allowed to react in the dark for 30 min (27). The absorbance of the solution was measured at 517 nm. Control biscuit samples (without *Forsythia suspensa* leaf powder, as described in Section 2.3.1) and vitamin C (VC) were used as blank and positive controls, respectively. All control samples were subjected to the same extraction and assay procedures as the test samples. The scavenging rate was calculated according to the following formula:


$$\begin{array}{l} DPPH\;free\;radical\;scavenging\;rate\;\left(\%\right)=\;\left(1-\frac{A1-A2}{A3}\right)\times 100 \end{array}$$

Among them, A_1_ represented the absorbance value of the sample + DPPH, A_2_ represented the absorbance value of the sample + ethanol, and A_3_ represented the absorbance value of ethanol + DPPH.

Determination of ABTS radical scavenging ability: the same sample solution extracted with ethanol was mixed with the ABTS color development solution and reacted for 5 min. The absorbance was measured at a wavelength of 734 nm. Control biscuit samples (without *Forsythia suspensa* leaf powder, as described in Section 2.3.1) and vitamin C (VC) were used as blank and positive controls, respectively. All control samples were subjected to the same extraction and assay procedures as the test samples. The calculation formula for the scavenging rate was:


$$\begin{array}{l} ABTS\;Free\;Radical\;Scavenging\;Rate\;\left(\%\right)=\left(\frac{A_{b}-A_{s}}{A_{b}}\right)\times 100 \end{array}$$

Among them, A_b_ represented the absorbance value of ethanol + ABTS, and A_s_ represented the absorbance value of sample + ABTS.

Determination of hydroxyl radical scavenging ability: the sample solution reacted with FeSO_4_, H_2_O_2_ and salicylic acid ethanol solution under a light protected condition at 37 °C for 30 min. The absorbance was measured at a wavelength of 510 nm. The absorbance was measured at a wavelength of 734 nm. Control biscuit samples (without *Forsythia suspensa* leaf powder, as described in Section 2.3.1) and vitamin C (VC) were used as blank and positive controls, respectively. All control samples were subjected to the same extraction and assay procedures as the test samples. The scavenging rate was calculated according to the following formula:


$$\begin{array}{l} R_{OH}=\left(1-\frac{A_{1}-A_{0}}{A_{a}}\right)\times 100\% \end{array}$$

Among them, A_1_ represented the absorbance value of the sample group, A_0_ represented the absorbance value of the blank group, and A_a_ represented the absorbance value of the control group.

### 2.4 Data analysis

All experiments were conducted in triplicate, and the results were expressed as mean ± standard deviation. Statistical analysis was performed using SPSS software (version 27.0, IBM Corp., Armonk, NY, USA). One-way analysis of variance (ANOVA) was applied to evaluate the differences among samples, followed by Duncan's multiple range test for multiple comparisons. Differences were considered statistically significant at *p* < 0.05. For multivariate analysis, principal component analysis (PCA) and partial least squares discriminant analysis (PLS-DA) were performed to distinguish among drying treatments, and key variables were selected based on variable importance in projection (VIP) values. Data visualization was carried out using Origin 2024 (OriginLab Corp., USA) and GraphPad Prism (version 10. 6, GraphPad Software, Boston, Massachusetts, USA). Different lowercase letters in tables and figures indicated statistically significant differences among treatments (*p* < 0.05).

## 3 Results

### 3.1 Effect of different *Forsythia suspensa* leaf drying methods on the volatile components of millet–*Forsythia suspensa* biscuits

#### 3.1.1 Overall flavor profile analysis based on electronic nose

**Figure 1:**
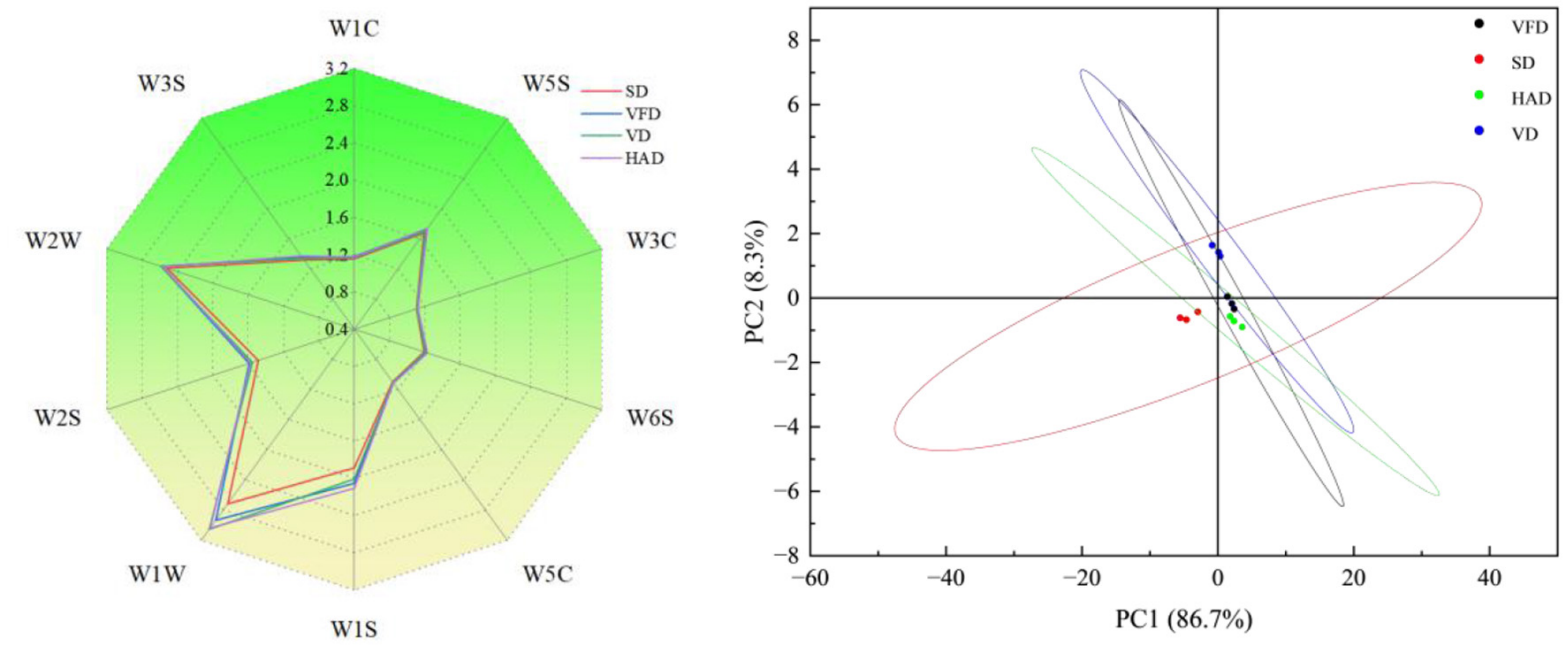
Effect of different *Forsythia suspensa* leaf drying methods on electronic nose responses and principal component analysis of volatile flavor characteristics in millet–forsythia biscuits.

As shown in Figure 1, significant differences in sensor responses were observed among the drying treatments. W1W exhibited the highest response intensity across all groups. HAD (3.038) and VFD (2.936) showed higher values than SD (2.717). For W1S, response values followed the order HAD (2.109) > VFD (2.055) > VD (2.009) > SD (1.890). W2W responses were slightly higher in VFD (2.581), VD (2.590), and HAD (2.564) compared with SD (2.522). The W5S sensor displayed the trend HAD (1.737) > VFD (1.729) > VD (1.699) > SD (1.676). Overall response intensity ranked as HAD ≈ VFD > VD > SD. Clear separation among drying treatments was observed based on the sensor response patterns. Principal component analysis further distinguished the samples. PC1 and PC2 explained 86.7 and 8.3% of the total variance, respectively, with a cumulative contribution of 95.0%. The high cumulative variance indicated that the model effectively represented the volatile differences among samples. However, some overlap was observed between the HAD and VFD samples in certain regions of the score plot, indicating that the two drying methods shared similarities in certain classes of volatile compounds detected by the E-nose sensors. These similarities may be associated with shared aroma-active compounds, such as aldehydes and alcohols derived from common precursors. Nevertheless, differences in the relative abundances and compositions of these compounds still contributed to the overall separation observed between the treatments (28). In the score plot, HAD samples were distributed along the positive direction of PC1. VFD and VD were located in the intermediate region but separated along PC2. SD samples clustered on the negative side of PC1 and were clearly separated from the other treatments.

#### 3.1.2 Overall distribution characteristics of volatile components based on GC-MS analysis

**Figure 2:**
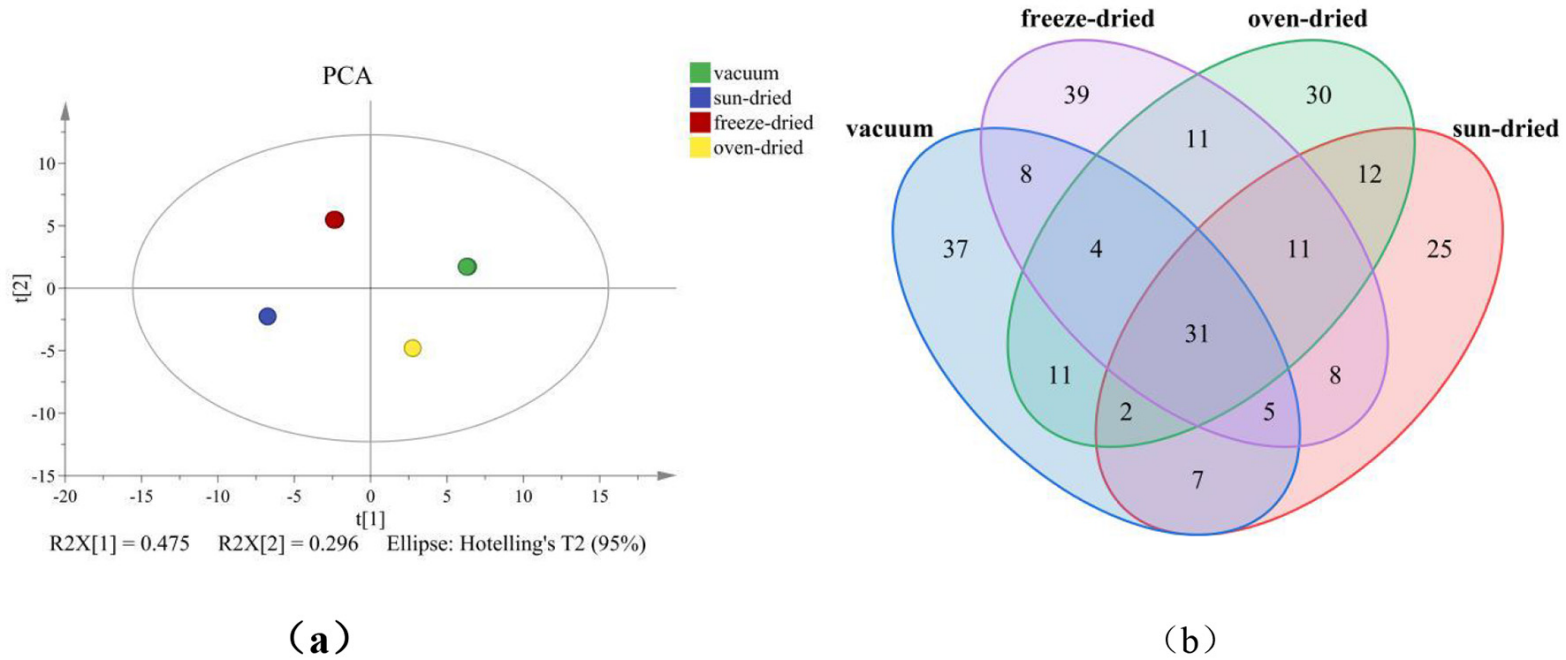
Effect of different *Forsythia suspensa* leaf drying methods on the overall distribution and characteristic differences of volatile components in millet–forsythia biscuits; **(a)** PCA analysis; **(b)** Venn diagram of volatile substances.

Figure 2a showed that the first two principal components explained 77.1% of the total variance (PC1 = 47.5%, PC2 = 29.6%). This level of cumulative variance adequately represented the overall differences in volatile composition among drying treatments. In the PCA score plot, the four sample groups were distributed in distinct quadrants without noticeable overlap. The spatial distribution followed a clockwise pattern: VFD, VD, HAD, and SD. Clear clustering of each treatment indicated that drying method markedly influenced the volatile composition.

Notably, the PCA results obtained from E-nose and GC–MS exhibited different variance contribution patterns. In the E-nose analysis, PC1 explained 86.7% of the total variance, indicating that the overall volatile intensity dominated the sensor responses. In contrast, GC–MS PCA showed that PC1 accounted for only 47.5% of the variance, suggesting a more complex and multidimensional volatile composition. This discrepancy was mainly attributed to the different detection principles of the two techniques. The electronic nose captured the overall aroma profile through sensor array signals, providing a comprehensive representation of the volatile characteristics. In contrast, GC–MS was used to separate and quantify individual volatile compounds, thereby offering detailed information on the chemical composition (29, 30). Therefore, GC–MS PCA reflected variations among multiple volatile components, resulting in a more balanced variance distribution across principal components. These findings indicated that E-nose and GC–MS provided complementary information for evaluating volatile profiles, with E-nose emphasizing overall odor intensity and GC–MS revealing detailed compositional differences (31).

Figure 2b illustrated the distribution of shared and unique volatile compounds under different drying conditions. A total of 31 compounds were common to all treatments, formed the basic aroma framework of the samples. VD exhibited 37 unique compounds, the highest among all groups. VFD contained 39 unique compounds. HAD and SD showed 30 and 25 unique compounds, respectively. Despite these differences, several compounds were shared between two or three treatments, suggesting that certain volatile components were independent of drying strategy.

#### 3.1.3 Screening of key differential compounds based on GC-MS fingerprint profiles

**Figure 3:**
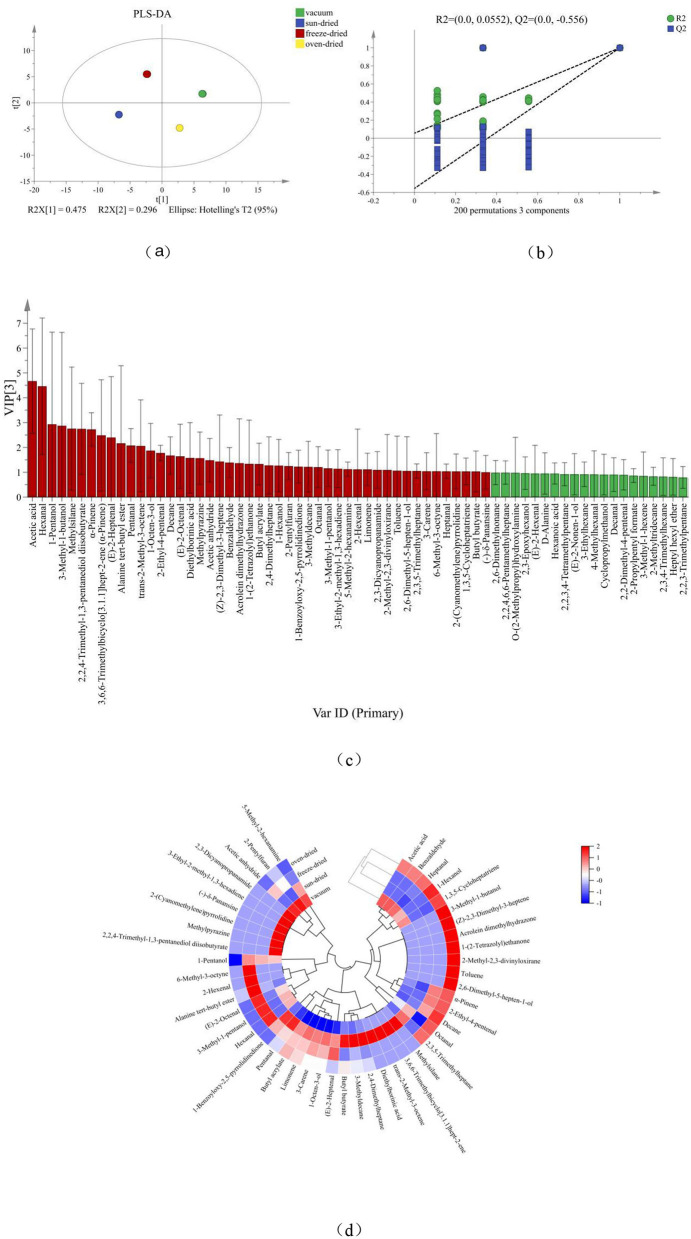
Effect of different *Forsythia suspensa* leaf drying methods on multivariate discrimination and key differential compound screening of volatile components in millet–forsythia biscuits; **(a)** PLS-DA score plot; **(b)** 200-time permutation test plot; **(c)** VIP score plot; **(d)** clustering heatmap of key differential compounds.

To enhance group discrimination, partial least squares discriminant analysis (PLS-DA) was performed on the volatile dataset. As shown in Figure 3a, the first two latent variables explained 77.1% of the total variance (R^2^X[1] = 0.475; R^2^X[2] = 0.296). All samples were located within the Hotelling's *T*^2^ (95%) confidence ellipse, and no outliers were detected. The four drying treatments occupied distinct regions in the score plot without noticeable overlap, confirming significant differences in volatile composition among treatments. Model robustness was evaluated using a 200 times permutation test (Figure 3b). The intercepts of *R*^2^ and *Q*^2^ were 0.0552 and −0.556, respectively. All permuted *Q*^2^ values were lower than that of the original model, and the regression line showed a clear downward trend. The negative *Q*^2^ intercept indicated that the model was not overfitted and possessed acceptable predictive reliability. Variable importance in projection (VIP) analysis identified key discriminant markers (Figure 3c). Among 54 detected volatile compounds, 47 exhibited VIP values >1, accounting for 87.0% of the total. These compounds were considered major contributors to sample differentiation. Only seven compounds showed VIP values < 1 and contributed minimally to classification.

The dominant markers included aldehydes (hexanal, pentanal, octanal, heptanal, (E)-2-heptenal, 2-hexenal, 2-ethyl-4-pentenal, benzaldehyde), alcohols (1-pentanol, 3-methyl-1-butanol, 1-octen-3-ol, 1-hexanol, 3-methyl-1-pentanol, 2,6-dimethyl-5-hepten-1-ol), terpenes (α-pinene, 3-carene, limonene, 3,6,6-trimethylbicyclo[3.1.1]hept-2-ene), and heterocyclic or nitrogen-containing compounds (methylpyrazine, 2-pentylfuran). Hexanal, acetic acid, and several unsaturated aldehydes ranked among the highest VIP values. In contrast, compounds such as 2,6-dimethylnonane, 2,2,4,6,6-pentamethylheptane, hexanoic acid, and D-alanine showed VIP values < 1 and limited discriminative contribution. A heatmap constructed from the 47 key markers (Figure 3d) revealed distinct expression patterns among treatments. HAD, VFD, SD, and VD samples formed distinct clusters, indicating that the regulation of volatile compounds depended on the drying treatment.

#### 3.1.4 Category composition analysis of key differential compounds identified by GC-MS

**Figure 4:**
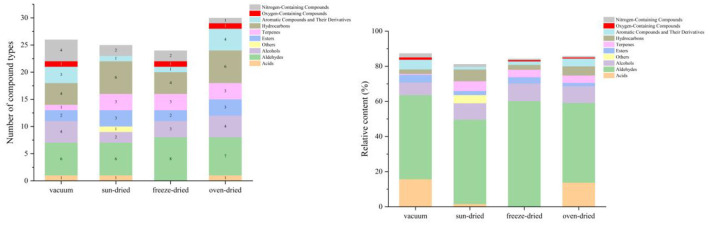
Effect of different *Forsythia suspensa* leaf drying methods on the composition of key differential volatile compounds in millet–forsythia biscuits.

As shown in Figure 4, distinct differences in volatile composition were observed among drying treatments. VD samples contained 26 differential compounds. Aldehydes (6) and alcohols (4) were dominant, while hydrocarbons (4) and ketones (2) were relatively balanced in distribution. SD samples contained 25 compounds. Aldehydes (6) and hydrocarbons (6) were present in relatively high numbers. Several esters and other minor compounds were also detected. VFD samples comprised 24 compounds, with aldehydes showing the highest count (8). In contrast, acids and nitrogen containing compounds were less abundant. HAD exhibited the greatest diversity, with 30 identified compounds. Aldehydes, hydrocarbons, and nitrogen containing compounds were increased compared to the other treatments. Regarding relative abundance, aldehydes were the predominant class across all drying methods. However, their proportional contribution varied. VFD showed the highest aldehyde proportion. In VD and HAD, acids accounted for a larger percentage than in SD and VFD. SD samples displayed relatively higher proportions of terpenes, hydrocarbons, and other minor components, with a more dispersed compositional structure.

### 3.2 Effect of different *Forsythia suspensa* leaf drying methods on the taste characteristics and flavor compounds of millet–*Forsythia suspensa* biscuits

#### 3.2.1 Overall taste profile analysis based on electronic tongue

**Figure 5:**
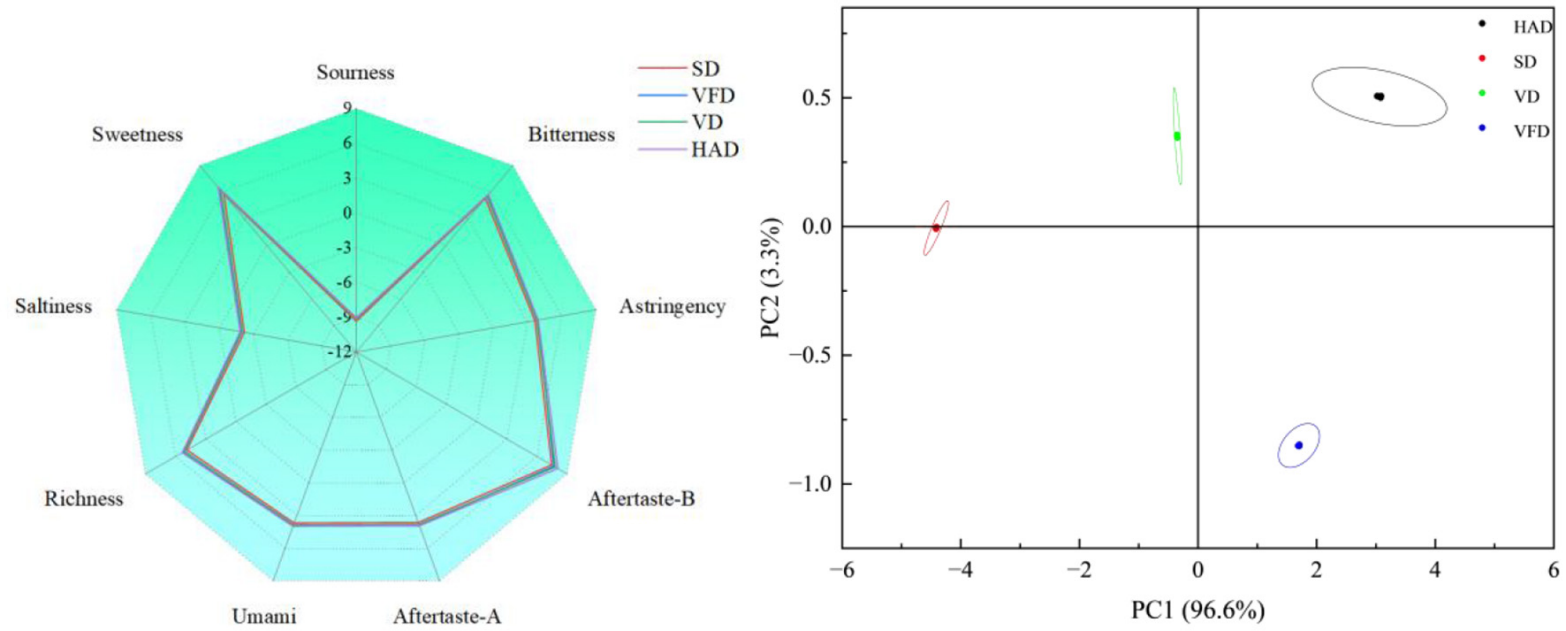
Effect of different *Forsythia suspensa* leaf drying methods on radar chart and principal component analysis of flavor characteristics in millet–forsythia biscuits as determined by electronic tongue.

As shown in Figure 5, the radar plot demonstrated a clear gradient in overall taste intensity among drying treatments, following the order: HAD > VFD > VD > SD. HAD samples exhibited the highest responses for most taste attributes, including bitterness (5.731), astringency (3.947), aftertaste-B (8.083), aftertaste-A (3.945), umami (3.989), richness (5.352), and sweetness (6.538). These values indicated a stronger taste perception and longer aftertaste persistence in the HAD group. VFD samples ranked second in umami (3.946) and richness (5.287), and showed higher values than SD (umami 3.694; richness 4.881). In contrast, SD samples presented the lowest responses across most taste dimensions, particularly in umami and richness. Sourness and saltiness responses were negative in all treatments (approximately −9.0 to −9.3 for sourness and −1.8 to −2.2 for saltiness), indicating lower relative responses compared with the reference solution. Among treatments, HAD showed the smallest absolute saltiness value (−1.836).

Principal component analysis revealed that PC1 explained 96.6% of the total variance, while PC2 accounted for 3.3%. The cumulative contribution rate reached 99.9%, indicating that the electronic tongue dataset effectively captured taste variations among treatments. PC1 clearly separated SD from HAD. VFD and VD were distributed between these two groups, reflecting intermediate taste intensity. PC2 provided limited additional discrimination, although a partial separation between HAD and VFD was observed. Replicates clustered tightly within each group, and confidence ellipses showed no evident overlap, confirming good repeatability and significant differences among drying treatments.

#### 3.2.2 Analysis of free amino acid composition and its contribution to flavor

As shown in Table 2, drying methods significantly affected the retention levels and taste composition of free amino acids (FAAs). Overall, VFD samples exhibited the highest concentrations in all taste related categories. Umami amino acids (UAA) reached 343.90 ± 3.41 μg/mL, with Glu as the dominant component (238.67 ± 2.48 μg/mL), followed by Asp (105.23 ± 1.16 μg/mL). Sweet amino acids (SAA) amounted to 516.72 ± 4.58 μg/mL, mainly contributed by Ala (147.22 ± 2.61 μg/mL) and Gly (126.24 ± 1.56 μg/mL). Bitter amino acids (BAA) showed the highest overall concentration (714.04 ± 4.62 μg/mL), with Leu (132.94 ± 1.83 μg/mL) and Lys (127.88 ± 2.21 μg/mL) as the predominant constituents. VD ranked second, with UAA, SAA, and BAA contents of 321.48 ± 2.71, 492.27 ± 3.41, and 665.33 ± 3.85 μg/mL, respectively. SD samples showed lower levels than VD but remained markedly higher than HAD. HAD treatment resulted in the lowest FAA concentrations. UAA decreased to 269.31 ± 3.20 μg/mL, SAA to 441.84 ± 3.87 μg/mL, and BAA to 570.23 ± 4.02 μg/mL. All treatments exhibited a consistent distribution pattern of BAA > SAA > UAA. Although the relative taste structure remained unchanged, the total retention levels varied significantly among drying methods.

**Table 2:** Effect of different *Forsythia suspensa* leaf drying methods on amino acid composition and taste classification of samples.

Taste category	Amino acids	Content (μg/mL)			
		SD	VFD	VD	HAD
Umami amino acids	Asp	91.53 ± 0.59^c^	105.23 ± 1.16^a^	97.82 ± 0.76^b^	83.18 ± 1.09^d^
	Glu	207.62 ± 1.90^c^	238.67 ± 2.48^a^	223.66 ± 2.09^b^	186.13 ± 2.51^d^
	Subtotal	299.15 ± 2.34^c^	343.90 ± 3.41^a^	321.48 ± 2.71^b^	269.31 ± 3.20^d^
Sweet amino acids	Ser	75.56 ± 1.33^c^	85.89 ± 1.34^a^	80.74 ± 1.58^b^	69.53 ± 1.28^d^
	Thr	70.94 ± 1.54^c^	82.48 ± 1.86^a^	76.76 ± 1.46^b^	64.31 ± 1.67^d^
	Gly	113.67 ± 2.43^c^	126.24 ± 1.56^a^	119.98 ± 1.69^b^	107.35 ± 1.43^d^
	Ala	135.42 ± 2.73^c^	147.22 ± 2.61^a^	141.31 ± 1.62^b^	129.53 ± 1.52^d^
	Pro	72.69 ± 1.51^c^	74.89 ± 1.27^a^	73.48 ± 1.18^b^	71.13 ± 1.46^d^
	Subtotal	468.28 ± 4.12^c^	516.72 ± 4.58^a^	492.27 ± 3.41^b^	441.84 ± 3.87^d^
Bitter amino acids	Val	97.73 ± 2.37^c^	111.66 ± 1.76^a^	104.42 ± 1.64^b^	91.01 ± 1.44^d^
	Met	48.00 ± 0.96^c^	57.27 ± 1.83^a^	51.56 ± 1.35^b^	45.84 ± 1.42^d^
	Ile	68.72 ± 1.60^c^	79.92 ± 1.95^a^	74.31 ± 1.66^b^	64.74 ± 1.28^d^
	Leu	115.33 ± 2.66^c^	132.94 ± 1.83^a^	123.93 ± 1.74^b^	108.94 ± 1.99^d^
	Phe	57.57 ± 1.48^c^	63.96 ± 1.76^a^	60.72 ± 1.34^b^	53.72 ± 1.33^d^
	His	44.92 ± 1.50^c^	52.11 ± 1.06^a^	48.54 ± 1.06^b^	42.21 ± 1.09^d^
	Arg	77.76 ± 1.59^c^	88.30 ± 2.59^a^	83.20 ± 1.74^b^	74.23 ± 1.81^d^
	Lys	108.63 ± 2.20^c^	127.88 ± 2.21^a^	118.66 ± 1.67^b^	89.54 ± 1.68^d^
	Subtotal	618.66 ± 5.04^c^	714.04 ± 4.62^a^	665.33 ± 3.85^b^	570.23 ± 4.02^d^
Other functional amino acids	Cys-Cys	21.55 ± 0.60^c^	26.99 ± 1.30^a^	24.21 ± 0.34^b^	20.23 ± 0.48^d^
	Tyr	39.83 ± 0.82^c^	46.34 ± 1.44^a^	42.69 ± 1.17^b^	37.52 ± 0.99^d^
	Subtotal	61.37 ± 1.34^c^	73.32 ± 2.13^a^	66.90 ± 1.35^b^	57.75 ± 1.24^d^

Values are expressed as mean ± standard deviation (n = 3).

Different lowercase letters within the same row indicate significant differences among drying treatments (*p* < 0.05).

### 3.3 Effect of different *Forsythia suspensa* leaf drying methods on the textural properties and microstructure of millet–*Forsythia suspensa* biscuits

#### 3.3.1 Analysis of textural properties (TPA) parameters

As shown in Table 3, drying methods significantly affected the textural properties of millet–*Forsythia suspensa* leaf biscuits (*p* < 0.05). For hardness, VD (22.97 N) and HAD (21.47 N) were significantly higher than SD and VFD. SD exhibited the lowest value (17.06 N), indicating a relatively loose structure. Fracturability followed a similar trend. VD showed the highest value (21.99 N), whereas SD was the lowest (11.37 N). This result suggested that vacuum drying promoted the formation of a structure with greater fracture resistance. Deformation at fracture differed among treatments. HAD displayed the highest value (1.45 mm), indicating greater deformation before breakage. SD showed the shortest fracture distance, reflecting a more brittle structure. For initial modulus, SD presented the highest value (41.39 N/mm), significantly exceeding HAD. This indicated higher rigidity at small deformation levels. In contrast, HAD samples were relatively softer at the initial compression stage. Elasticity was also significantly influenced by drying method. HAD exhibited the highest value (0.69 mm), whereas VFD showed the lowest. Chewiness demonstrated the most pronounced difference. HAD reached the highest value (3.70 mJ), followed by VD. SD and VFD remained at comparatively lower levels.

**Table 3:** Effect of different *Forsythia suspensa* leaf drying methods on texture properties of millet–forsythia biscuits.

Parameter	Unit	SD	VFD	VD	HAD
Hardness	N	17.06 ± 0.83^c^	18.10 ± 0.91^b^	22.97 ± 1.35^a^	21.47 ± 1.12^a^
Fracturability	N	11.37 ± 0.92^c^	17.80 ± 1.21^b^	21.99 ± 1.76^a^	18.55 ± 1.48^b^
Deformation at fracture	mm	0.27 ± 0.03^c^	0.58 ± 0.04^b^	0.66 ± 0.05^b^	1.45 ± 0.12^a^
Initial modulus	N/mm	41.39 ± 4.86^a^	33.48 ± 3.72^ab^	27.36 ± 2.98^b^	11.85 ± 1.42^c^
Springiness	mm	0.56 ± 0.04^b^	0.26 ± 0.02^c^	0.55 ± 0.03^b^	0.69 ± 0.05^a^
Chewiness	mJ	0.50 ± 0.06^c^	0.10 ± 0.02^c^	1.70 ± 0.19^b^	3.70 ± 0.41^a^

Values are expressed as mean ± standard deviation (n = 3).

Different lowercase letters within the same row indicate significant differences among drying treatments (*p* < 0.05).

#### 3.3.2 Microstructural characterization based on scanning electron microscopy

**Figure 6:**
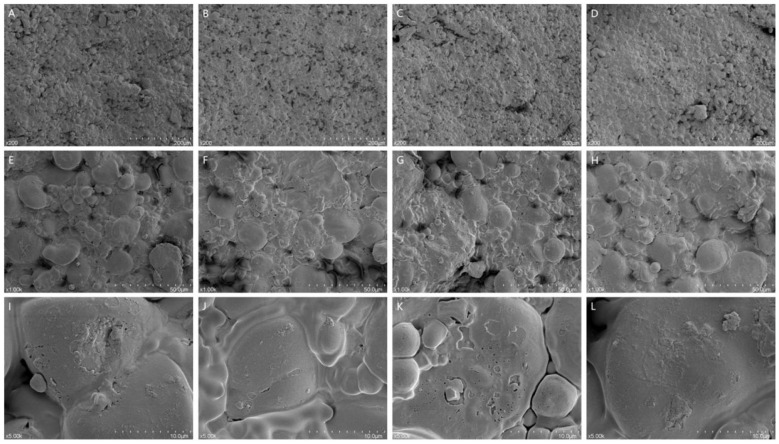
Effect of different *Forsythia suspensa* leaf drying methods on the morphology of millet–forsythia biscuits. **(A, E, I)** SD; **(B, F, J)** VFD; **(C, G, K)** VD; **(D, H, L)** HAD.

As illustrated in Figure 6, drying methods markedly influenced the microstructure of millet–*Forsythia suspensa* leaf biscuits. At the 200 μm scale, SD samples exhibited a relatively uniform matrix with a rough surface and irregular pore distribution. VFD samples showed a loose and porous architecture, with increased pore number and more homogeneous distribution. VD samples appeared more compact, with localized aggregation regions. HAD samples displayed large pores and partial structural collapse. At 50 μm magnification, partial exposure of starch granules was observed in SD samples, although the matrix remained tightly packed. VFD samples showed clearly defined starch granules and evident interfacial separation, indicating low structural compactness. VD samples exhibited enhanced granule fusion and the formation of a more continuous phase. In contrast, HAD samples showed pronounced granule coalescence and matrix embedding. At 10 μm resolution, SD samples presented microcracks and a relatively rigid structure. VFD samples showed interconnected internal pores and a loose interface. VD samples maintained a more integrated gelatinized starch morphology with fewer fissures. HAD samples exhibited surface shrinkage and localized collapse, suggesting rapid moisture migration during high-temperature drying.

### 3.4 Effect of different *Forsythia suspensa* leaf drying methods on functional active component content and antioxidant capacity of millet–*Forsythia suspensa* biscuits

**Figure 7:**
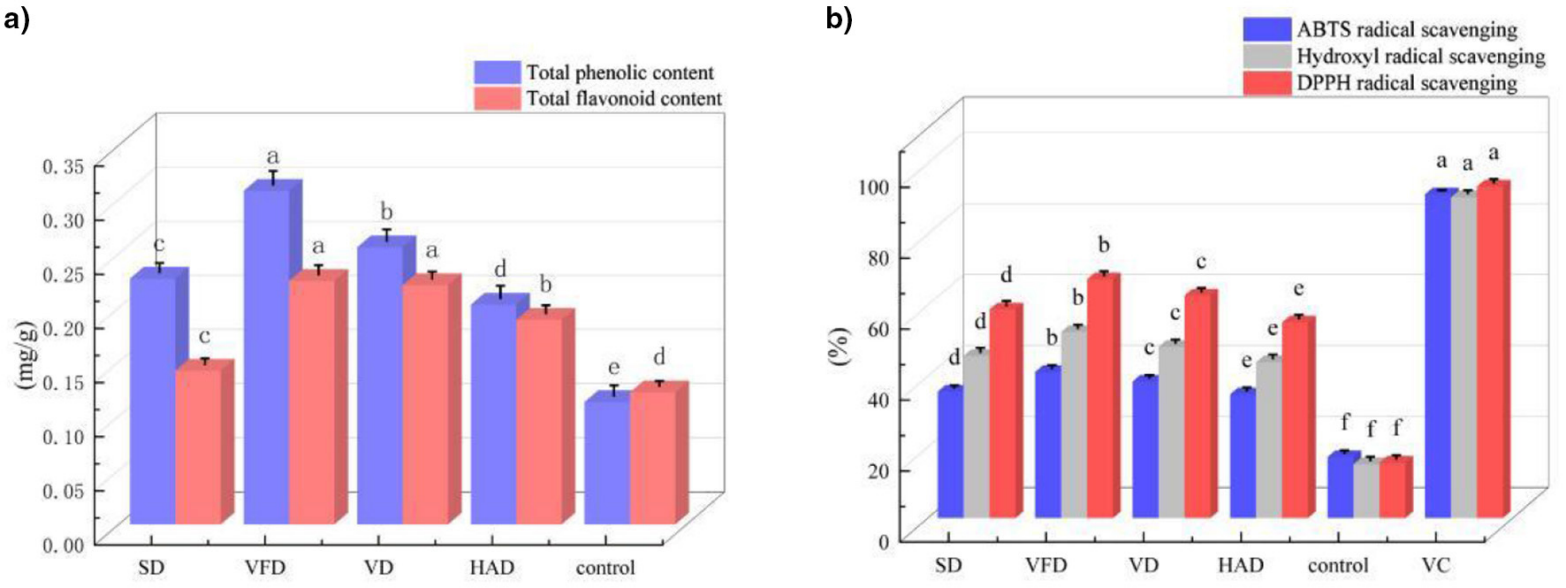
Effect of different *Forsythia suspensa* leaf drying methods on the functional active components and antioxidant capacity of millet–forsythia biscuits. **(a)** Total phenolic and flavonoid contents; **(b)** Radical scavenging activities. Different superscript letters indicate significant differences, *p* < 0.05.

As shown in Figure 7a, drying methods significantly affected the retention of phenolic compounds in millet–*Forsythia suspensa* lea f biscuits (*p* < 0.05). Among all treatments, VFD samples exhibited the highest total phenolic content (TPC), followed by VD, SD, and HAD, while the control biscuit showed the lowest value. A similar trend was observed for total flavonoid content (TFC). VFD and VD samples did not differ significantly in TFC (*p* > 0.05), but both were significantly higher than SD, HAD, and the control. These results indicated that vacuum freeze drying and vacuum drying were more effective in preserving phenolic substances than hot-air drying and sun drying.

Figure 7b further showed that drying treatments significantly influenced antioxidant activity (*p* < 0.05). For all three radical scavenging systems (ABTS, hydroxyl radical, and DPPH), the positive control (VC) exhibited the highest scavenging capacity, whereas the blank biscuit (control) showed the lowest activity. Among the four drying treatments, VFD consistently showed the strongest antioxidant capacity, followed by VD, SD, and HAD. Notably, SD samples exhibited significantly higher ABTS, hydroxyl radical, and DPPH scavenging activities than HAD, indicating that hot air drying caused greater losses of antioxidant components. Although all biscuits samples showed lower scavenging activities than VC, the addition of *Forsythia suspensa* leaves markedly improved the antioxidant capacity compared with the control biscuit. Overall, the variation in antioxidant activity was consistent with the changes in TPC and TFC. The superior performance of VFD may be attributed to its low temperature and low pressure conditions, which better preserved antioxidant substances during processing.

## 4 Discussion

The present study demonstrated that different drying methods significantly affected the volatile profile, taste characteristics, microstructure, and antioxidant properties of millet–*Forsythia suspensa* leaf biscuits. These differences likely were attributed to variations in thermal intensity, oxygen exposure, and mass transfer conditions during drying. Such factors were likely to influence key chemical pathways, including lipid oxidation, Maillard reactions, structural transformation of macromolecules, and the degradation of antioxidant compounds.

GC-MS analysis revealed that aldehydes were the dominant differential volatile compounds among the drying treatments. In particular, hexanal, octanal, and (E)-2-heptenal were significantly enriched in the hot air drying (HAD) group. These aldehydes were typical secondary products of lipid oxidation and were widely associated with fatty, green, and roasted aroma notes in thermally processed foods. Their higher abundance likely indicated that high temperature drying promotes lipid oxidation reactions. According to the lipid oxidation mechanism described by Fereidoon, lipid hydroperoxides formed during primary oxidation are unstable and can decompose into secondary oxidation products. Under thermal conditions, they likely decomposed into aldehydes, ketones, and other volatile compounds (32–34). In addition, elevated temperature likely enhanced radical formation and promoted the propagation of lipid oxidation chain reactions. Reactive oxygen species (ROS) generated during heating may further accelerate lipid degradation (35). Choe and Min reported that an increase in temperature significantly enhanced ROS production and accelerated the conversion of lipid hydroperoxides into secondary oxidation products (35). This mechanism likely helped explain the pronounced accumulation of aldehydes observed in the HAD samples. Similar phenomena have been reported in thermally processed plant based foods. For example, hot air drying has been shown to increase lipid derived volatile compounds and intensify roasted aroma characteristics (36). Besides lipid oxidation, high temperature likely also promoted Maillard reactions between amino acids and reducing sugars. These reactions were considered important pathways for the formation of roasted aroma compounds in baked products. Nitrogen containing heterocyclic compounds, such as pyrazines, were typical products of the Maillard reaction and contributed strongly to nutty and roasted flavor notes (37, 38). Li et al. (39) emphasized that the Maillard reaction was highly temperature dependent and became increasingly dominant in thermally processed food systems. Therefore, the stronger roasted aroma intensity detected in the HAD samples was likely associated with the combined effects of lipid oxidation and the formation of Maillard derived volatiles.

Recent studies have reported similar influences of drying temperature on flavor development in plant based materials. Wu et al. (40) demonstrated that hot air drying significantly increased lipid oxidation derived volatile compounds and enhanced the overall aroma intensity of dried pepper products. However, the same study also reported a reduction in polyphenol content and antioxidant capacity after thermal drying. This finding highlighted a common phenomenon in thermal food processing: flavor formation was often enhanced at the expense of functional compounds. In contrast, vacuum freeze drying (VFD) was performed under low temperature and reduced pressure. These conditions likely limited oxygen exposure and suppressed thermal degradation reactions. As a result, the VFD samples retained a higher diversity of volatile compounds in this study. The ability of vacuum freeze drying to preserve aroma compounds has been widely documented. Fellows noted that vacuum freeze drying minimizes heat induced degradation and volatile losses because sublimation occurs at low temperatures (41). In addition, Li et al. (42) showed that freeze dried mushrooms contained more volatile compounds and exhibited a more complex aroma profile than hot air dried samples. These observations agreed well with the results obtained in the present study. Electronic tongue analysis and amino acid quantification further indicated that high temperature drying increased overall taste intensity while significantly reducing the levels of free amino acids. This result suggested that amino acids likely participated in non-enzymatic browning reactions or thermal degradation processes. During thermal processing, amino acids likely were involved in Maillard reactions or undergo Strecker degradation. These pathways likely generated various volatile flavor compounds. Peng reported that such reactions converted amino acids into aldehydes, heterocyclic compounds, and melanoidins, which reduced their original concentration in food matrices (43).

From a kinetic perspective, Boekel demonstrated that the rate constant of the Maillard reaction increased exponentially with temperature (44). This kinetic behavior likely explained the simultaneous enhancement of flavor signals and the decrease in amino acid content observed in the HAD samples. Therefore, the stronger taste responses detected by the electronic tongue were likely partly due to newly formed Maillard reaction products rather than the original amino acid composition. Microstructural analysis further showed that drying methods significantly influenced the structural organization of the biscuit matrix. Heat treatment during hot air drying was likely to promote starch gelatinization and protein cross linking. These changes likely resulted in a denser network structure and increased mechanical strength. Rutigliano described that thermal processing likely induced protein unfolding, aggregation, and intermolecular interactions, ultimately forming stable protein networks in food systems (45). Interactions between gelatinized starch and aggregated proteins may further strengthen the structural matrix of baked products. In contrast, the VFD samples exhibited a highly porous and interconnected structure. This structure likely originated from ice crystal formation and sublimation during freeze drying. Freeze dried products typically displayed porous microstructures due to the removal of ice crystals during sublimation (46). Such porous structures likely helped retain volatile compounds and improve aroma preservation. However, they likely also reduced mechanical strength, resulting in lower hardness compared with thermally dried products. The changes in antioxidant activity observed in this study closely followed the variation in polyphenol content. In addition, it was noted that *Forsythia suspensa* contains unique bioactive compounds, including phenylethanoid glycosides (e.g., forsythiaside), lignans (e.g., phillyrin and pinoresinol), and other secondary metabolites (7). These compounds were known to exhibit strong antioxidant and pharmacological activities (47). Previous studies have suggested that these components may respond differently to processing conditions than general plant polyphenols, due to their distinct chemical structures and stability characteristics (48). However, the specific behavior of these compounds under different drying conditions was not directly investigated in this study and warrants further research. This result suggested that phenolic compounds may play an important role in antioxidant capacity. However, antioxidant activity in complex food matrices was not solely determined by phenolic compounds. It may also be influenced by synergistic interactions among various bioactive compounds, the presence of other antioxidants such as vitamins and Maillard reaction products, as well as possible alterations in antioxidant assay responses caused by processing conditions. Similar considerations were reported in previous studies (33, 49). Processing temperature and oxygen exposure were key factors affecting the stability of phenolic compounds. Wu et al. reported that thermal processing reduced the antioxidant potential of plant based foods, primarily due to the degradation of polyphenols and other redox active molecules (50). Snoussi et al. also reported that high temperature drying reduced total phenolic content and antioxidant capacity in dried plant materials (51).

Overall, the results suggested that drying methods may influence product quality through several interconnected mechanisms. Thermal intensity and oxygen availability regulated lipid oxidation and Maillard reaction kinetics, which may determine the formation of volatile aroma compounds. At the same time, these conditions affected amino acid stability, polyphenol degradation, and macromolecular structural transformations. The combined effects of these reactions ultimately determined the balance between flavor formation, structural properties, and nutritional functionality in millet–*Forsythia suspensa* leaf biscuits. An understanding of these mechanisms provided useful theoretical guidance for optimizing drying processes in plant based functional baked products. Future studies should integrate flavor chemistry, structural characterization, and nutritional evaluation to better control product quality during thermal processing.

## 5 Conclusion

This study systematically investigated how four drying methods, namely natural sun drying (SD), vacuum freeze drying (VFD), vacuum drying (VD), and hot air drying (HAD), affect the flavor characteristics, taste active amino acid profile, microstructure, texture properties, and antioxidant activity of millet-*Forsythia suspensa* leaf biscuits. Multidimensional analytical techniques, including GC–MS, electronic nose, electronic tongue, and scanning electron microscopy, were used to clarify the mechanisms underlying quality variation during drying. The results showed that drying methods significantly affected the composition and abundance of volatile compounds, while HAD and VFD exhibited stronger electronic nose responses than VD and SD. GC-MS combined with PLS-DA identified 47 key differential compounds (VIP > 1), among which aldehydes and alcohols were the main contributors. This indicates that lipid oxidation and Maillard reactions were intensified under higher drying temperatures. Taste analysis further revealed that HAD generated the strongest overall taste intensity, whereas VFD retained the highest levels of taste-active amino acids. Structural observations showed that VFD formed a uniform porous network structure, while VD and HAD produced denser matrices with greater mechanical strength. In addition, VFD preserved higher total phenolic content and antioxidant capacity compared with high temperature drying treatments. Overall, VFD showed clear advantages in retaining volatile compounds and bioactive substances, whereas HAD and VD were more conducive to the development of stronger instrumental flavor intensity and a more compact product structure. These findings provide valuable insights for optimizing drying strategies in plant based functional baked products, as well as for balancing flavor development, structural stability, and nutritional functionality.

## Data Availability

The raw data supporting the conclusions of this article will be made available by the authors, without undue reservation.
